# Animal Models for Heart Transplantation Focusing on the Pathological Conditions

**DOI:** 10.3390/biomedicines11051414

**Published:** 2023-05-10

**Authors:** Horng-Ta Tseng, Yi-Wen Lin, Chun-Yao Huang, Chun-Ming Shih, Yi-Ting Tsai, Chen-Wei Liu, Chien-Sung Tsai, Feng-Yen Lin

**Affiliations:** 1 Taipei Heart Institute, Taipei Medical University, Taipei 11031, Taiwan; 2 Division of Cardiology and Cardiovascular Research Center, Taipei Medical University Hospital, Taipei 11031, Taiwan; 3 Departments of Internal Medicine, School of Medicine, College of Medicine, Taipei Medical University, Taipei 11031, Taiwan; 4 Institute of Oral Biology, National Yang Ming Chiao Tung University (Yangming Campus), Taipei 112304, Taiwan; 5 Division of Cardiovascular Surgery, Tri-Service General Hospital, Defense Medical Center, Taipei 11490, Taiwan; 6 Department of Basic Medical Science, College of Medicine, University of Arizona, Phoenix, AZ 85721, USA; 7 Department and Graduate Institute of Pharmacology, National Defense Medical Center, Taipei 11490, Taiwan

**Keywords:** heart transplantation, animal models

## Abstract

Cardiac transplant recipients face many complications due to transplant rejection. Scientists must conduct animal experiments to study disease onset mechanisms and develop countermeasures. Therefore, many animal models have been developed for research topics including immunopathology of graft rejection, immunosuppressive therapies, anastomotic techniques, and graft preservation techniques. Small experimental animals include rodents, rabbits, and guinea pigs. They have a high metabolic rate, high reproductive rate, small size for easy handling, and low cost. Additionally, they have genetically modified strains for pathological mechanisms research; however, there is a lacuna, as these research results rarely translate directly to clinical applications. Large animals, including canines, pigs, and non-human primates, have anatomical structures and physiological states that are similar to those of humans; therefore, they are often used to validate the results obtained from small animal studies and directly speculate on the feasibility of applying these results in clinical practice. Before 2023, PubMed Central^®^ at the United States National Institute of Health’s National Library of Medicine was used for literature searches on the animal models for heart transplantation focusing on the pathological conditions. Unpublished reports and abstracts from conferences were excluded from this review article. We discussed the applications of small- and large-animal models in heart transplantation-related studies. This review article aimed to provide researchers with a complete understanding of animal models for heart transplantation by focusing on the pathological conditions created by each model.

## 1. Introduction

Heart transplantation is the final therapeutic option for the treatment of heart failure. Patients undergoing heart transplants face significant complications due to transplant rejection. Animal experiments are necessary to study the mechanisms of disease onset and develop treatments for these complications. Several animal models have been developed to investigate different research topics, including the immunopathology of graft rejection, immunosuppressive therapies, anastomotic techniques, and graft preservation techniques. The application of these studies’ findings and recommendations to clinical practice has led to significant increases in graft survival rate and improved overall patient prognosis. Researchers must understand the advantages and limitations of each animal model and select the appropriate model for their research objective to advance the research process efficiently and provide clinical references for precise treatments [1]. Various small and large animal models are reviewed to integrate the concept of multiple animal models with heart transplantation, and the applicable research objectives, advantages, and disadvantages of each model are discussed.

Rodents, rabbits, and guinea pigs are frequently used in heart transplantation experiments [1]. The use of small animals in these experiments is advantageous because of their smaller size, higher metabolic rate, higher reproductive rate, ease of handling, and lower cost [1,2,3]. Because there are numerous genetically well-characterized strains of rats and mice, these small animals have been used as model organisms in most heart transplantation studies. Using transgenic or gene knockout strains with clear genetic information permits the creation of specific pathological environments and the simulation of specific rejection responses, such as cell- or antibody-mediated alloreactive immune responses and rejection-induced vasculopathy. These models enable researchers to stimulate the rejection of a cell- or an antibody-mediated alloreactive immune response and elucidate the molecular mechanisms of rejection after transplantation to develop effective interventions [2,3]. Large-animal model organisms, including canines, pigs, and non-human primates, rather than small animal model organisms, are used for distinct research purposes [1]. Primarily, small animals are used to test hypotheses in pathological research and develop therapeutic modalities. However, these findings rarely translate directly to clinical applications. Large animals have anatomical structures and physiological states similar to those of humans; therefore, large-animal models are often used to validate the results of small-animal studies and determine the clinical applicability of the results. Research on immunosuppressive agent development, heart transplantation techniques, and the preservation of donor grafts frequently use large-animal models. In the heart transplantation field, large-animal models play an important role in preclinical trials and the development of clinical translational applications [1,3].

In this review article, we conducted a literature search on animal models for heart transplantation, focusing on the pathological conditions using PubMed Central^®^ at the United States National Institutes of Health’s National Library of Medicine before 2023. The unpublished reports and abstracts from conferences were excluded from this review article. The applications of small- and large-animal models in heart transplantation-related studies were discussed, with a focus on the pathological conditions established by each model to provide researchers with a more comprehensive understanding of heart transplantation animal models.

## 2. Small-Animal/Rodent Models

Animal models for heart transplantation include orthotopic transplantation and heterotopic transplantation models. Due to the small size of rodents, orthotopic transplantation is extremely difficult to perform and therefore not preferred by most researchers; consequently, heterotopic transplantation is primarily discussed in this review section (Table 1).

### 2.1. Heterotopic Heart Transplantation in Mice

In mouse abdominal heterotopic cardiac transplantation [28,29], the inferior vena cava (IVC) and pulmonary vein of the donor heart are ligated, and the donor aorta and pulmonary artery are sutured end-to-side to the recipient abdominal aorta and IVC, respectively. The advantages of this model include its simple operation, reproducibility, and effective oxygenation of the donor graft [30]. However, the left ventricle (LV) of the transplanted heart does not beat spontaneously and lacks hemodynamics, leading to alterations in cardiac physiology, myocardial degeneration, and atrophy. The outcome of heterotopic cardiac transplantation may deviate from actual clinical circumstances [2,31]. Based on the mouse abdominal heterotopic heart transplantation model, Klein et al. implanted balloons in the LV of the donor graft to maintain the isovolumic work of the heart to reduce the damage caused by the absence of LV work in the donor graft [32]. Furthermore, Spencer et al. proposed a puncture aortic valve model to increase LV pressure via aortic regurgitation [31], which alleviated some of the effects of the lack of LV pressure pulsation.

#### 2.1.1. Models Exploring the Role of Innate Immune System in Heart Transplantation

Matzinger et al. reported that signals induced by damaged tissue may play a greater role in activating the immune response than in recognizing foreignness [33]. Additionally, they emphasized that innate immunity is essential for distinguishing self/non-self patterns and mediating the immune response [34]. Consequently, the relationship between innate immune response and allograft transplant rejection has emerged as an essential area of investigation in organ rejection. Numerous models have been developed to investigate the tissue damage caused by cardiac allograft transplantation, including ischemic-reperfusion injury and subsequent rejection, posttransplant infection, and resulting damage-associated molecular patterns (DAMPs) [35,36]. Innate immune cells include monocytes, macrophages, neutrophils, natural killer (NK) cells, platelets, and NK T cells. These models allowed us to understand the role of the innate immune system in allotransplant rejection and its interaction with the adaptive immune system.

##### Roles of Toll-like Receptors (TLRs) and DAMPs (Table 2)

The innate immune system responds to sequences exhibited by pathogens or damaged cells, including carbohydrates and lipid moieties [41]. TLRs play critical roles in these responses. TLRs are mainly expressed in antigen-presenting cells (APCs) such as macrophages, monocytes, dendritic cells, NK cells, T cells, and B cells, and endothelial, epithelial, and smooth muscle cells also express TLRs. TLRs primarily serve to recognize pathogens or DAMPs [35,41]. After recognition by TLRs, pathogens or DAMPs activate the intracellular downstream signaling pathway and regulate innate immunity-induced inflammatory responses [36,42,43,44]. Previous studies reported that TLRs affect long-term graft outcomes after heart transplantation. Organ harvesting is often accompanied by prolonged cold ischemia and ischemia/reperfusion (I/R) injury after blood flow reperfusion, which stimulates the release of DAMPs from cells. Oyama et al. and Arslan et al. used C57BL/10 ScCr *TLR4*^−/−^ and C57BL/6 *TLR2*^−/−^ mice, respectively, to study the issue. Their results demonstrated that TLR2 and TLR4 are positively correlated with the occurrence of I/R injury [37].

Researchers have also analyzed the interaction between TLR-induced downstream molecular pathways and cardiac allograft rejection. I/R injury induces necrosis in donor allografts, and necrotic cells release danger signals that induce metabolic changes in innate immune cells [45,46,47]. Activated monocytes and macrophages secrete the danger-signaling, high-mobility group box 1 (HMGB1) protein, which is passively released by necrotic or damaged cells. Additionally, HMGB1 binds to TLR4 on the macrophages and innate immune cells, inducing downstream inflammatory responses [38,39,47,48]. Zhu et al. transplanted heart-infiltrating cold Bretschneider solution from C57BL/6 mice into C57BL/6 *TLR4*^−/−^ mice to verify the role of the HMGB1-TLR4-interleukin (IL)-23-IL-17A axis in cardiac transplant-induced I/R injury [38]. Additionally, they used glycyrrhizin, an HMGB1 inhibitor in the C57BL/6 mouse heart, in a syngeneic C57BL/6 mouse model to understand the regulation of the HMGB1-TLR4 axis on the expression of IL-23 and IL-17A after transplantation. Huang et al. used a C57BL/6 mouse heart heterotopically transplanted into a BALB/c mouse as a research model. They found that the HMGB1 is passively released by injured tissues or actively secreted by graft-infiltrating innate immune cells. Additionally, they used A-box, a specific antagonist of HMGB1, to investigate the effect of inhibiting HMGB1 on graft survival rate [39]. The intracellular signals induced by TLRs on APCs after antigen recognition usually activate myeloid differentiation factor 88 (MYD88), a common adapter protein [49,50]. Transplanting BALB/c *MyD88*^+/+^ mouse heart or BALB/c *MyD88*^−/−^ mouse heart into a C57BL/6 mouse is a commonly used model for investigating the roles of MYD88 in heart transplantation [40].

##### Roles of Monocytes, Macrophages, Neutrophils, and NK Cells (Table 3)

Innate immune cells are crucial in initiating an immune response to allografts. Clinical data has shown that most cells infiltrating the transplanted organ during allograft rejection are macrophages [60,61,62]. Chronic rejection is proportional to the intensity of macrophage infiltration in grafts [63,64]. In the chronic allograft vasculopathy lesions of a patient with chronic heart transplant rejection, macrophages were observed [65]. Kitchens et al. used a murine heterotopic cardiac transplantation model to deplete macrophages. C57BL/6 mouse hearts were transplanted into (C57BL/6 mouse × BALB/c mouse) F1 recipients. In this study, we administered carrageenan intraperitoneally to deplete macrophages without affecting T, B, or NK cells. Macrophage depletion reduces the incidence of allograft vasculopathy. Antimacrophage therapy may assist in elucidating the effects of macrophages on cardiac allograft vasculopathy [51]. Monocyte recruitment and accumulation are critical in rejection and cardiac allograft vasculopathy. Valenzuela et al. injected donor-specific major histocompatibility complex (MHC) I antibodies into C57BL/6 *RAG1*^−/−^ recipients with BALB/c cardiac allografts to induce antibody-mediated rejection (AMR). P-selectin upregulation in cardiac allograft endothelial cells and macrophage infiltration into cardiac allografts were then analyzed. Furthermore, they used rPSGL-1, a selectin antagonist, to determine the effect of P-selectin inhibition on monocyte infiltration into allografts [52]. In addition to clinical models of macrophage-mediated rejection, models of the different phenotypes and functions of polarized macrophages are discussed.

Macrophages are versatile effector cells that exhibit a high degree of phenotypic plasticity and develop into functionally diverse subsets in response to environmental stimuli or other immunological effector cells. Activated macrophages are classified within a spectrum of polarization states, with the M1-polarized phenotype (classically activated) and M2-polarized phenotype (alternatively activated) representing the two extremes of the spectrum. Interferon-γ (IFN-γ) and lipopolysaccharides stimulate the M1-polarized phenotype, while IL-4 or IL-13 induces the M2-polarized phenotype. Macrophages exposed to other stimuli can be categorized based on their phenotypic similarity to M1-polarized or M2-polarized macrophages [56,66,67,68]. Zhao et al. targeted the pivotal signaling molecules tumor-necrosis factor receptor-associated factor 6 (TRAF6) and mammalian target of rapamycin (mTOR), which orchestrate macrophage polarization into M1 and M2, respectively. They transplanted complete-MHC-mismatched BALB/c mouse hearts into C57BL/6 mice, LysM^Cre^Traf6^fl/fl^ C57BL/6 mice (non-TRAF6 expressing), and LysM^Cre^Mtor^fl/fl^ C57BL/6 mice (non-mTOR-expressing) to examine the effects of TRAF6 and mTOR on macrophage differentiation during allograft rejection. Furthermore, they elucidated a coinhibitory role in graft rejection by inhibiting the PD-1/PD-L1 pathway in LysM^Cre^Mtor^fl/fl^ C57BL/6 cardiac recipients [53]. Braza et al. transplanted the hearts of BALB/c mice into C57BL/6 mice or C57BL/6 mice without colony-stimulating factor 1 (CSF1) expression and induced allograft tolerance by injecting anti-CD40L mAb. They aimed to determine the role of CSF1 expression in macrophage polarization during allograft rejection.

Additionally, CSF1 may contribute to the formation of suppressive Ly6C^lo^ (M2) macrophages during tolerance induction. Braza et al. used Fucci transgenic mice as heart transplantation recipients to study the cell cycle and proliferative potential of Ly6Clo macrophages during tolerance [54]. Braza et al. designed a model that depleted regulatory Ly6C^lo^ M2 in a heart transplant recipient. BALB/c hearts were transplanted into C57BL/6 CD169 diphtheria toxin receptor recipient mice, and Ly6Clo M2 was depleted by administration of diphtheria toxin. This model was used to investigate the mechanism of action of mTOR in regulatory macrophages (Mreg) that produce graft tolerance [55].

In addition to the states of macrophage polarization previously identified, mouse monocytes exposed to macrophage CSF and IFN-γ were differentiated into a suppressive phenotype known as Mreg. Mouse Mregs are novel macrophage polarization states distinct from M1 and M2 subsets and monocyte-derived dendritic cells [55,56]. Riquelme et al. utilized a complete MHC mismatch heart heterotopic transplant model, transplanting C3H mouse hearts into BALB/c mice and C57BL/6 mouse hearts into BALB/c mice. They intravenously injected the donor strain Mregs into recipient mice before transplantation to examine the effects of Mregs on prolonging the survival of heart grafts. Mregs derived from B6.129P2-*Nos2^tm/Lau^*/J mice were also used to investigate the role of inducible nitric oxide synthase in Mreg action in cardiac allograft rejection [56].

Neutrophils participate in innate immune responses after organ transplantation. El-Sawy et al. investigated the impact of graft infiltration by polymorphonuclear leukocytes (PMNs) in graft rejection. A/J mouse hearts were heterotopically transplanted into CXCR2-antisera-treated C57BL/6 or *CXCR2*^−/−^ C57BL/6 mice. The PMN-attractant chemokines were depleted to study PMN infiltration and expression of proinflammatory cytokines in grafts. The researchers developed models to investigate the synergistic effects of PMN depletion and costimulation blockade [57]. In addition to T and B cells, NK cells mediate alloimmune responses to MHC heterogeneity. Uehara et al. established a semiallogeneic cardiac transplantation model to investigate the role of NK cells. They combined C57BL/6 mouse hearts with C57BL/6 mouse × BALB/c mouse F1 recipients, C57BL/6 mouse hearts with C57BL/6 mouse x B10.D2 mouse F1 recipients, and C57BL/6 mouse hearts with C57BL/6 mouse × C3H/HeJ mouse F1 recipients in this semiallogenic model without the use of immunosuppressive agents. This model may have induced cardiac allograft vasculopathy [58]. In another study, Hirohashi et al. injected donor-specific antiNK1.1 mAbs into a C57BL/6 *RAG1*^−/−^ mouse that had received a heart transplant from a B10.BR mouse. This prevented the development of cardiac allograft vasculopathy and demonstrated the importance of NK cells in chronic allograft rejection [59].

##### Roles of Complement Activation

Complement activation is an important component of the innate immune system, with higher activity during the early stages of transplantation, including I/R injury and hyperacute and acute rejection [69]. Like TLRs, complement proteins may function as pattern recognition receptors mediating innate immunity. Studies indicate that the complement system contributes to the induction of I/R injury in a mouse model [42,43]. Moreover, the complement system affects adaptive immunity. Uncontrolled activation of the complement cascade leads to enhanced T-cell reactivity and promotes allograft rejection [70]. The complement-related cardiac allograft transplant models will be discussed further in the “Models for AMR” section.

#### 2.1.2. Models of Alloimmunity Determined by Major Histocompatibility Complex (Table 4)

MHC disparity typically results in alloimmunity in mouse heart transplantation models, and it is an important issue for adaptive immune response. Based on the differences in research objectives and designs, researchers can select from a complete MHC disparate transplantation model, a single class I/II MHC mismatch model, or a minor histocompatibility antigens mismatch model to meet the research requirements for the intensity of rejection. Additionally, researchers can also select appropriate models for specific rejection reactions to further elucidate the rejection mechanism after heart transplantation.

Corry et al. and Schenk et al. have proposed fully MHC-disparate mouse abdominal heterotopic heart allograft transplant models. Corry et al. used the heart of an A/J mouse as the donor and the offspring of 129 and C57BL/10 (B10) mice as the recipient; this transplantation combination produced MHC disparity in H-2K, H-2D, and multiple non-H-2, eliciting an alloimmune response [28]. Schenk et al. grafted an A/J (H-2a) mouse heart to the abdominal aorta of a C57BL/6 (H-2b) mouse [20,71]. Other strains (including C57BL/10, DBA/1 interbreed, and C3H mice; BALB/c (H-2d) and C57BL/6; and C3H (H-2k) and BALB/c mice) have been used to develop a fully MHC-disparate model to investigate specific immune rejection pathways [20,56]. These fully MHC-disparate models develop rejection between 7 and 10 days after transplantation, analogous to the time course of clinical acute cell-mediated rejection in humans. These models elicit an obvious, rapid, and intense rejection response. However, they do not allow the researcher to properly distinguish the differences between the molecular mechanisms of rejection produced by different MHCs.

Results of studies using single class I or class II MHC mismatch models enable researchers to understand the roles of MHC and T cells in complex rejection reactions. Corry et al. developed the class I MHC mismatch model using B10.D2 and B10.BR mice as donors and transplanted the hearts into the abdominal aortas of the F1 hybrid mice between C57BL/6 and A/J mice. This combination caused a mismatch between the H-2K and H-2D regions on the class I MHC, resulting in an immune response to the specific antigen in the recipient mouse [28]. Schenk et al. developed a class I MHC mismatch model using the BALB/c mouse [71] by first introducing CD8-positive T cells reactive to Ld (class I MHC antigen) into a Rag1 gene knockout C57BL/6 mouse (which lacks mature B or T cells) and transplanting the heart of the Ld-expressing BALB/c mouse to its abdominal aorta. This combination allows the recipient to generate a rejection reaction against the H-2Ld (haplotype of BALB/c mouse strain’s class I MHC) expressed on the BALB/c mouse heart graft.

However, transplantation of the heart of a non-Ld-expressing DBA/1 mouse does not induce a rejection reaction in the recipient, allowing the study of immune rejection induced by class I MHC mismatches [20]. Schenk et al. also transplanted the heart of BALB/c (H-2dm2) mice (without the expression of H-2Ld) or Ld-expressing BALB/c mice to the abdominal aortas of C57BL/6 mice. This combination was used to investigate the effect of class I MHC expression on rejection. Hattori et al. developed a class I MHC mismatch model by transplanting the hearts of Kd-expressing C57BL/6 mice into the abdominal aortas of wild-type C57BL/6 mice [72].

Additionally, a class II MHC mismatch model has been developed. Researchers transplanted the heart of a B6C.H-*2^bm12^* mouse into the abdominal aorta of a wild-type C57BL/6 mouse. B6C.H-*2^bm12^* is a spontaneous mutation of the 1-Ab molecule that results in a 3-aa substitution in the third hypervariable region of the A-beta chain that differs from that of wild-type C57BL/6 mice at the 1-A locus of class II MHC but is identical at class I MHC and minor MHC loci [73,74]. This model can be used to study acute rejection and rejection-induced cardiac allograft vasculopathy [21,76].

Minor mismatched histocompatibility antigens (mH-Ags) also contribute to rejection reactions in the recipient, in addition to MHC mismatch-induced rejection [18,19]. In a related study, Corry et al. developed a minor mH-Ags model by transplanting the heart of a 129/J mouse into the abdominal aorta of a C57BL/10 mouse. This combination induced multiple non-H-2 disparities and rejection reactions [28]. Using these models, researchers can compare the rejection induced by mH-Ags to that induced by a complete MHC mismatch and analyze the distinctions in rejection and immune mechanisms predominant between the two. Kwun et al., who performed abdominal heterotopic heart transplantation with a donor BALB/c mouse (H-2d) and a recipient C57BL/6 mouse (h-2b) and a donor BALB.B mouse (H-2b) and a recipient C57BL/6 mouse (h-2b), respectively, used this method to determine the differences in reaction speed between mH-Ags-mediated and complete MHC mismatch-mediated heart graft rejection [19]. Sho et al. performed abdominal heterotopic heart transplantation using a donor C57BL/6 (H-2b) mouse with a recipient BALB/c (major mismatch) mouse and a donor B10.D2 mouse with a recipient BALB/c mouse (minor mismatch) [75]. He et al. transplanted a heart from a male C57BL/6 mouse into the abdominal aorta of a female C57BL/6 mouse [18] to examine the relationship between T cell-mediated rejection and mH-Ags mismatch.

In conclusion, the MHC mismatch model is a fundamental technique used by researchers to understand cardiac allotransplantation rejection. Transplantation between distinct strains of rats or between mutant, inbred, or gene-modified animal strains generates specific rejection responses. The appropriate selection of models allows for the effective and precise control of the intensity of the immune response induced by transplantation and the analysis of related rejection and pathological mechanisms.

#### 2.1.3. Models for Antibody-Mediated Rejection

In addition to cell-mediated rejection, antibody-mediated rejection has a significant impact on recipient outcomes and the longevity of transplanted organs. Therefore, murine abdominal heterotopic cardiac transplantation models with antibody-mediated allograft rejection have been developed for transplantation-related studies [77,78,79].

Researchers must first identify the target antigens on the transplanted heart graft, then administer the donor antigen-reactive monoclonal antibody directly into the recipient mouse; this is a common method of establishing an antibody-mediated rejection model. Most studies use anticlass I MHC antibodies [4,5,60,80,81,82]. Furthermore, antibody-mediated complement activation during antibody-mediated rejection contributes to graft injury by causing diffuse complement deposition (C4d and C3d) on the vascular endothelium and graft arteriopathy. Therefore, antibody-mediated rejection models are often used to examine the effects of complement and antidonor antibodies on xenografts or allografts. Selecting different monoclonal antibodies according to the research objective is essential in this model. Hancock et al. used immunoglobulin (Ig) G and IgM [60], while Rahimi et al. and Ding et al. used IgG2 (complement activating antibody) and IgG1 (non-complement activating antibody) to investigate the relationship between complement and rejection [4,82]. The greatest advantage of the antibody-mediated rejection model induced by antibody injection is that the researcher can demonstrate the effect of the antigen (such as MHC or anti-α1,3-galactosyltransferase [αGal]) on the donor graft rejection response based on the immune pathway being studied. However, this approach has limitations. Because monoclonal antibodies recognize only a single epitope, the rejection response is weak. A higher concentration of two or more different antibodies is needed to generate a strong rejection response [83]. Consequently, some researchers have used polyclonal antibodies to induce more effective rejection reactions [6]. Additionally, this model has been predominantly used in studies involving antibodies to class I MHC, although human endothelial cells express class II MHC molecules that murine cells lack. Therefore, the role of class II MHC in antibody-mediated rejection cannot be studied using this model [84,85].

Nozaki et al. investigated the relationship between antibody concentration and rejection by injecting the serum of sensitized animals into unsensitized animals. They transplanted the hearts of A/J (H-2a) mice into the abdominal aortas of C57BL/6 *CCR5*^−/−^ or wild-type C57BL/6 mice, then collected the sensitized serum, which was injected into C57BL/6 *RAG-1*^−/−^ mice that received A/J mouse heart transplantation. This study showed that the intensity of rejection induced by heart transplantation into C57BL/6 *RAG-1*^−/−^ mice is related to the titer of donor-specific antibodies and is caused by the serum of sensitized C57BL/6 *CCR5*^−/−^ with higher antibody titers than those in wild-type C57BL/6 mice [7]. Similar models have been used to examine the effect of Igs on rejection reactions [7,86]. In summary, this model can compensate for some of the shortcomings of the antibody-induced rejection model.

Previous studies have reported that antibody-mediated rejection is the cause of hyperacute rejections after organ transplantation, and antibody-induced complement activation is another major cause of hyperacute rejections [5,8,9,11,12]. αGal antibodies regulate complement-dependent hyperacute rejection; therefore, Galα1-3Galβ1-4GlcNAc has been used as an antigen to stimulate hyperacute rejections. Ding et al. and Yin et al. used rat-to-mouse abdominal xenotransplantation models in their studies, with αGal-expressing Lewis baby rats as the heart donors and C57BL/6 mice, which do not express αGal, as the recipients [5,8,9]. Shimizu et al. used a 129/C57BL/6 mouse (H-2b) as the heart donor and a C57BL/6 *GalT*^−/−^ (H-2b) mouse unable to produce αGal as the recipient [10]. Gock et al. injected rabbit red blood cell membranes into BALB/c *αGal*^−/−^ mice to induce anti-αGal antibodies [11,12] and subsequently transplanted the hearts of BALB/c *αGal*^+/+^ mouse into their abdominal aortas [12]. All of these models induced hyperacute rejection reactions. Allograft and xenograft transplantation models are common surgical procedures used to investigate hyperacute rejection. Because there is no difference in histocompatibility between donors and recipients in the allograft transplantation model, the interactions between the αGal epitope and anti-αGal antibodies can be studied independently. In addition to their function in the rejection and immune response, alloantibodies can induce the adaptation of the cardiac allograft and play a protective role in certain circumstances in patients undergoing organ transplantation [8,82,87]. Ding et al. developed a model of graft accommodation by transplanting a baby Lewis rat heart, together with repeated intravenous injection of anti-α-Gal IgG1, into a *Rag/GT*-deficient mouse [8]. The results of these studies indicate that antibody pretreatment may confer protective effects on the graft. The donor graft’s vascular endothelium may express genes that protect the graft from antibody-mediated rejection and improve its survival.

Murine antibody-induced rejection models have some limitations. First, murine and human hearts have significant anatomical differences [88,89]. In humans, the coronary arteries are located primarily on the surface of the heart and are embedded in epicardial fat. However, murine coronary arteries are only on the surface of the heart when they emerge from the aortic root, and most of the vessels cross the myocardium into the intramural space. However, antibodies rely primarily on blood vessels transported to the graft to generate a subsequent inflammatory response. Therefore, variations in vascular structure and pathways between species may result in different rejection responses and outcomes. In humans, chronic allograft vasculopathy develops in the epicardium after a heart transplant due to rejection reactions, though this is less common in intramural models [88,89]. Rejection in murine heart transplantation models occurs more often in the intramural area of the heart, leading to medial necrosis and fibrosis [90]. Additionally, human coronary arteries are supplied with oxygen and nutrients by the vasa vasorum, which is absent in murine models. During antibody-mediated rejection, these small vessels may allow alloreactive immune cells and inflammatory cytokines to be more widespread in the coronary arteries of the transplanted heart. The rejection induced by this phenomenon differs from that in murine models [1,91]. These limitations should be considered when investigating clinical translation applications.

Successful desensitization has improved the survival rate of organ transplants; however, AMR induced by human leukocyte antigen (HLA) incompatibility between the donor and recipient remains a major obstacle to successful allografting. Scientists have gained a deeper understanding of the mechanisms of AMR, the characteristics of anti-HLA antibodies, and gene expression profiles of allografts after more than 20 years of research [92]. Recent studies have shown that plasma exchange and immunoglobulin injection can effectively prevent AMR. Moreover, many small-animal preclinical tests have shown that the IgG-degrading enzyme of *Streptococcus pyogenes* (Ides), complement and proteasome inhibitors, anti-CD20 treatment, and IL-6 receptor blockers hold the potential to be applied in AMR management [93].

#### 2.1.4. Models That Induce Cardiac Allograft Tolerance

Immunosuppressive agents are the most direct method to induce tolerance of the donor graft in the recipient. In addition to medication, researchers have developed several animal models of graft tolerance in the recipient for use in relevant studies. The injection of blood or blood cells from an allogeneic donor into a recipient can induce tolerance for a specific allograft. Kishimoto et al. [13], Hancock et al. [14], and Burns et al. [15] have used this method to induce allograft tolerance and prevent chronic allograft rejection by infusing the recipient with donor splenic leukocytes and B7/CD154 signaling pathway inhibitors during organ transplantation. This method prolongs the survival and usability of the allograft. This model has been extensively used to examine the immune regulatory mechanism in cardiac allograft rejection, and this concept has been implemented in large-animal xenotransplantation models. Kwun et al. [16] and Kaplan et al. [17] injected a human CD52 transgenic mouse with alemtuzumab (a monoclonal antibody that binds to CD52) and induced lymphocyte depletion, rendering the immunocompromised recipient unable to reject the donor heart due to the development of graft tolerance.

Over the past decade, regulatory T cells (Tregs) have emerged as key mediators of immune stability. A study investigating Treg cell therapy in mice found that pretreatment with donor alloantigen and anti-CD4 mAb promoted the generation of CD4+CD25+ T cells in vivo, which effectively regulated skin allograft rejection [94]. Humanized mouse models have also demonstrated the potential of ex-vivo expanded human Tregs in preventing vasculopathy, skin rejection, and islet rejection after transplantation [95,96,97]. This model has demonstrated that different Treg populations have distinct regulatory efficacies and migration patterns that correlate with Treg function [98,99]. Therefore, targeting Tregs for transplant tolerance holds promise for patients undergoing organ transplantation.

### 2.2. Models for Regulating Allograft Rejection Using Skin Transplantation

Previous studies have reported that rejection reactions induced after heart and skin transplantation differ in intensity and pathological features. Skin grafts with MHC-mismatched or mH-Ags disparities typically induce acute rejection reactions [76,100], whereas cardiac rejection reactions are less likely to occur under the same donor–recipient combinations [75,101]. Thus, He et al. [18] transplanted the heart of a male C57BL/6 mouse into the abdominal aorta of a female C57BL/6 mouse and then transplanted the skin of a male C57BL/6 mouse onto a female C57BL/6 mouse 30 days later. This method enhanced the antimale T cell immunity of the female C57BL/6 mouse. The results suggest that cardiac grafts may induce an immune response in the recipient that is insufficient for the acute rejection of the heart graft. However, this response leads to chronic graft rejection and tissue damage. Boosting the immune response via skin transplantation in heart transplant recipients may hasten the development of a chronic graft injury; however, it is insufficient to cause an acute graft injury. Kwun et al. hypothesized that the rejection reactions induced by skin grafts differ from those induced by cardiac grafts, suggesting that the heart is promptly perfused after heart transplantation (a phenomenon known as primary vascularization). However, in skin transplantation, new blood vessels must form before the skin can receive adequate reperfusion [19]. To test this hypothesis, a heart and skin transplantation model with a minor MHC disparity was developed using a BALB/b donor and C57BL/6 recipient. A nonvascularized ear–pinna cardiac allograft transplantation model was used as a control. The results confirm that primary vascularization determines the immune dominance of mH-Ags in the rejection response. To investigate the interacting roles of immune regulatory cells and donor-reactive memory T cells during rejection, Schenk et al. transplanted class II MHC mismatched donor skin to a recipient with a long-term-surviving heart allograft (class II MHC mismatched) [20,21]. Sensitized immune cells from a C57BL/6 mouse transplanted with skin from an A/J mouse were transplanted into another C57BL/6 mouse with a heart from an A/J mouse to investigate the contribution of memory cells to transplant rejection [20].

### 2.3. Models of Combined Abdominal Heterotopic Heart and Aorta/Carotid Artery Transplantation

Cardiac allograft vasculopathy (CAV) is a sign of chronic rejection due to atherosclerosis of the transplanted heart’s blood vessels [102,103]. CAV was previously investigated using a mouse aortic transplant model [104]. Despite fully MHC-mismatched aortic allografts for transplantation and the absence of any immunosuppressive agents in this model, aortic allografts do not induce acute rejection in the animal, as opposed to clinical rejection. Therefore, the model’s translatability is limited [22,105]. A combined heart and aortic artery transplantation model have been developed to overcome these limitations. This model involves two major types of surgeries. First, the recipient’s left internal carotid artery was replaced with a donor carotid artery, and heterotopic abdominal heart transplantation was performed [22]. Then, a heart and abdominal aorta transplantation was performed heterotopically [23]. Because CAV is associated with acute cardiac parenchymal rejection after transplantation, combined aorta/carotid artery allograft and heart allograft transplantation may result in more significant intimal hyperplasia in CAV models and more closely resemble the actual pathological progression of CAV [22,23].

### 2.4. Heterotopic Aortic Transplantation in Rats

CAV is a major contributor to chronic graft failure caused by chronic rejection after heart transplantation. Heterotopic or orthotopic aortic transplantation is a common model used to investigate the CAV mechanisms [24]. The abdominal or thoracic aorta can be used as the donor graft. In this model, the aortic graft can be directly anastomosed to the recipient’s abdominal aorta. Donors and recipients can be matched based on major histoincompatibility, such as Brown Norway (RT1^n^) rats to Lewis (RT1^1^) rats, Lewis (RT1^1^) rats to Fischer (RT1^1^) rats, DA (RT1^a^) rats to Wistar–Firth (WF, RT1^a^) rats, or PVG/Seac (RT1^c^) rats to ACI/NKyo (RT1^a^) rats. Aortic grafts from Lewis rat, Fischer rat, and WF rat donors often induce acute vascular rejection in the recipient after transplantation; the long-term outcomes may differ from the vasculopathy caused by chronic rejection [24].

However, aortic transplantation in the PVG/Seac rats to ACI/NKyo rats model is more appropriate for studies regarding chronic rejection-induced allograft vasculopathy as the ACI/NKyo rat develops a weak acute rejection response against the graft aorta from the PVG/Seac rat. Lin et al. transplanted a thoracic aorta from a PVG/Seac rat to the abdominal aorta of an ACI/NKyo rat to study the endothelial mesenchymal transition as a mechanism that induces CAV and identify treatments for CAV by monitoring cell–cell interactions [25,26,27]. In addition, the authors used this method to investigate molecular pathways that may have protective effects against chronically-rejected grafts [27]. The aortic transplantation model using PVG/Seac rats and ACI/NKyo rats is simple. It provides a comprehensive understanding of the endothelial environment during chronic allograft vasculopathy [24]. However, rat heterotopic aortic transplantation is limited because the results do not translate well into the allograft vasculopathy of a whole harvested heart [24].

### 2.5. Limitations of Rodent Models

Rodents are suitable model organisms in the field of heart transplantation research, though there are some limitations. The immune systems of rodents are structurally and functionally different from the human immune system [3,106]. Furthermore, experimental rats are grown in a relatively pathogen-free environment, depriving their immune systems of pathogen stimulation. Therefore, the results obtained using these models differ from those of actual clinical patients [106,107]. Furthermore, immunosuppressive agents and modulators regulate and inhibit rejection after heart transplantation in patients. However, the use of immunosuppressive agents in rodent models of heart allograft transplantation is either non-existent or limited to subclinical levels. Therefore, the differences in the absorption and metabolic rate of medications between species must be considered when interpreting the results [108,109,110]. These limitations prevent the direct clinical application of the results of rodent studies, and preclinical trials must be bridged to more suitable species, including pigs and non-human primates.

## 3. Large-Animal Models (Table 5)

Large animals, including canines, pigs, and non-human primates, share anatomical structures and physiological functions that are similar to those of humans. Therefore, these animals are often used as model organisms in organ transplantation, especially in preclinical trials involving heart transplantation. During the early stages of heart transplantation research, canines were commonly used, though pigs and non-human primates are currently used more commonly. The cardiovascular structures of pigs closely resemble those of humans. The low cost and genetic manipulability of pigs have led to several preclinical trials related to heart transplantation and the development of allogeneic transplants. However, the left hemiazygos vein of pigs directly perfuses the coronary sinus; therefore, it must be ligated to avoid post-transplant bleeding [1,124]. Furthermore, as the right atrium and pulmonary artery tissue are less ductile in pigs, they can be easily torn during graft suturing. The heart is prone to arrhythmias, which must be considered when performing heart transplantation in pigs [124].

### 3.1. Orthotopic Heart Transplantation

Orthotopic heart transplantation models involve transplanting the donor heart into the recipient’s heart. Since 1994, several researchers have used 40–50 kg male Yorkshire pigs as donors and recipients. The surgical procedure in this model is similar to that in humans, and the recipient requires a cardiopulmonary bypass to support blood circulation and respiration during surgery. A biatrial anastomotic technique is used to suture the left and right atria of the donor heart to the posterior wall of the recipient’s atrium and to prevent the development of arrhythmias in the transplanted heart [111,112,113]. This model is primarily used to study the development of rejection and antirejection therapies after heart transplantation. It is a reliable preclinical model that links basic research results with practical clinical applications, as the findings obtained in small-animal models must be corroborated in this model before being translated to clinical trials. Additionally, as the porcine heart is very sensitive to ischemia, this model is suitable for studies regarding reperfusion injury, donor graft preservation strategies, and acute rejection [124].

### 3.2. Heterotopic Heart Transplantation Model

Unlike orthotopic heart transplantation, heterotopic heart implantation involves implanting the donor heart into a site in the recipient that differs from the normal site, such as the neck, abdomen, or thoracic cavity. Different implantation sites can be used according to the specific research objectives. In large animals, heterotopic transplantation is classified into non-working and working heterotopic heart transplantations.

#### 3.2.1. Non-Working Heterotopic Heart Transplantation Model

In the non-working heterotopic heart transplantation model, the implanted donor heart cannot support the recipient’s hemodynamic system. This heart transplantation model is primarily used to study the immunopathological changes caused by rejection or during the development of immunosuppressive agents/modulators. However, the implantation site differs from the original site of the heart, resulting in altered hemodynamic pressure on the myocardium or the absence of the natural pulsation of the myocardium, limiting the non-working heterotopic heart transplantation model. Early non-working heterotopic heart implantation studies were primarily conducted in canines. Carrel et al. transplanted the hearts of young dogs into the shoulders of mature dogs, combining the aorta, pulmonary artery, vena cava, and pulmonary veins of the donor’ heart with the external jugular vein and common carotid artery of the recipient. However, their work was unsuccessful as the cardiac chambers filled with thrombi [114,115]. Mann et al. refined Carrel’s model by connecting the canine donor heart’s aorta and pulmonary artery to the recipient’s common carotid artery and external jugular vein, thereby establishing coronary arterial circulation in the donor heart. The vena cava and pulmonary veins attached to the donor heart were ligated [114]. This technique overcame the obstacles of thrombus formation in the cardiac chambers and the hypoperfusion of the myocardium. This successful ligation approach has been applied to porcine and non-human primate models of heterotopic heart transplantation. For example, Michler et al. transplanted the hearts of outbred cynomolgus monkeys into outbred baboons. First, one of the mitral valve leaflets was excised after removing the heart of the cynomolgus monkey, and the foramen ovale was incised in the atrial septum. Next, the cynomolgus monkey aorta and pulmonary artery were connected to the baboon’s carotid artery and internal jugular vein. This technique maintains myocardial circulation and prevents thrombus formation, poor LV emptying, and incomplete LV filling [116]. Heterotopic transplantation of animals in which the donor heart is implanted into the neck of the recipient allows easier postoperative management than orthotopic transplantation because a chest drainage tube is not required since the thoracic cavity was not opened.

Furthermore, as the abdominal cavity is not opened, there is no obstruction after surgery [114,115]. The evaluation of the donor heart is also much simpler, as the tissue implanted in the graft can be easily harvested for biopsy without thoracotomy or laparotomy. The immune and inflammatory responses of animals undergoing heterotopic transplantation in the neck are relatively weak due to the absence of major trauma, and study outcomes tend to be more stable [116]. However, the volume of the donor heart that can be implanted into the subcutaneous tissue of the neck is limited.

Transplantation of a heart graft to the neck limits the size of the graft and may compress the pharynx of the recipient. Therefore, the abdominal cavity is also an implantation site for heterotopic transplanted models. Ohmi et al. removed the interatrial septum and tricuspid valve of a canine donor heart. They sutured the graft aorta to the abdominal aorta of the recipient and the graft superior vena cava to the IVC of the recipient. This approach allows for the maintenance of partial pulsatile pressure in the LV and simulates the working environment of the heart, although the blood flow to the donor heart is minimal [117]. Additionally, heterotopic heart transplantation using the porcine abdomen as the transplantation site was conducted. Hsu et al. selected 4–6-month-old Taiwanese Lanyu miniature pigs as donors and recipients of MHC-incompatible heart transplantation. The mitral valve of the donor heart was disrupted, and a hole was made in the atrial septum. Then, the donor’s ascending aorta and pulmonary artery were sutured to the recipient’s abdominal aorta and IVC, respectively. This model allows for sufficient pressure in the LV to prevent atrophy and thrombus formation [118]. Abdominal heterotopic transplantation overcomes the limitations of neck heterotopic transplantation. However, puncture biopsy for analyzing abdominal heterotopic transplantation in animals is associated with a high mortality rate [119].

Minanov et al. selected the retroperitoneum as a heterotopic cardiac transplantation site for the heart of a Yorkshire piglet into the retroperitoneum of a 3–7-week-old baboon, anastomosing the aorta and pulmonary artery of the donor heart to the common iliac artery and common iliac vein, respectively, of the baboon [119]. Additionally, thoracic implantation is also used in heterotopic heart transplantation models. Jamieson et al. used outbred mongrel dogs weighing 12–20 kg as the donor and recipient. Consistent with previous studies, the foramen ovale on the atrial septum of the donor heart was opened, and the aorta and pulmonary artery of the donor were anastomosed with the left innominate artery and superior vena cava, respectively [120].

Although using the abdomen or retroperitoneum as the graft site is more favorable, its susceptibility to complications, including postoperative ileus, intestinal obstruction, and intussusception, must be considered [120]. Non-working heterotopic heart transplantation models result in emptying of the LV, inadequate loading of the pumping fluid, and loss of normal function of the myocardial cells occur within a few days after the procedure [125], which severely limits this model.

#### 3.2.2. Working Heterotopic Heart Transplantation Model

Working heart transplantation models are necessary to maintain the transplanted heart close to the normal physiological environment and obtain similar results to clinical conditions. Demikhov et al. created a canine intrathoracic heterotopic heart transplant model that allows the LV of the donor heart to continue to contract and achieve a working load even after transplantation [121]. Furthermore, Losman et al. developed a biventricular support heterotopic cardiac transplant model that allows the left and right ventricles to be functional. They selected two adult chacma baboons of similar weight matched by ABO blood grouping as the donor and recipient. The left and right atria of the donor heart were anastomosed to the recipient’s left and right atria, respectively, and the aorta and pulmonary artery of the donor heart were anastomosed to the aorta and pulmonary artery, respectively. This model allows the donor heart to have loading stress that acts as a biventricular assist device [122].

Uwe et al. used Yorkshire pigs to develop a biventricular working model that could be used to compare the changes in the LV in non-working and working conditions. They anastomosed the left pulmonary artery of the donor heart to the left atrial appendage of the recipient prior to implantation. The aorta and right pulmonary artery of the donor heart were then anastomosed to the recipient’s descending aorta and infrarenal IVC, respectively. Researchers created a donor heart working model by blocking the right pulmonary artery; however, right ventricle blood flow was directed into the recipient’s IVC by blocking the left pulmonary artery and depriving the LV of the preload of the right heart, which led to left heart atrophy [123].

In the non-working heart transplantation model, atrial septal defects are often created and the atrial-ventricular valve is often disrupted before implanting the donor heart into the recipient. These changes cause the venous return of the recipient to flow to the right ventricle of the donor heart after transplantation. The blood returns to the left atrium, and ultimately flows through the recipient’s aorta during contraction. Therefore, non-working models in which the donor heart is pretreated may be considered partially or monoventricular working models [116,118,120].

### 3.3. Heart Xenotransplantation Model

Allograft heart transplantation is currently the only treatment method for patients with end-stage heart failure. However, allogenic donor hearts are inadequate to meet clinical needs; therefore, xenotransplantation has been proposed as a possible solution that requires further research. Xenotransplantation of pig hearts has potential, though several challenges remain [126,127,128].

The pig-to-primates non-working heterotopic heart xenotransplantation model has been used previously to study immune and rejection reactions after transplantation and the survival rate of the grafted heart. Several studies using this model have reported that the most important factors affecting graft and animal survival are the immunobiological barriers. Different immunosuppressive agents have been developed to modulate the various immune responses that occur in xenograft rejection. In practice, these methods allow for effective control of xenograft rejection. These rejection prevention methods may be applied to orthotopic xenograft transplantation and implemented clinically in the future [115,126,127,128]. Heart xenotransplantation models, similar to non-working heart heterotopic transplantation models, primarily involve transplanting the donor heart into the abdominal region of the recipient and anastomosing the aorta and pulmonary artery of the donor to the recipient’s abdominal aorta and IVC, respectively [115,129]. Early studies included transplanting the donor heart to the neck or retroperitoneal region of the recipient, though space restrictions limited the selection of the transplant site.

Galactose-α-1,3-galactose (Gal) is expressed in grafts, and anti-Gal antibodies present in the blood of non-human primate recipients lead to the activation of complement, resulting in humoral hyperacute rejection. These changes lead to acute failure of the graft and the subsequent failure of the pig-to-non-human primate xenotransplantation model. In 2005, Kuwaki et al. transplanted a non-Gal expressing α1,3-galactosyltransferase gene knockout porcine heart into baboons and recorded lower anti-Gal antibody levels in the blood. Furthermore, the expression of complement-regulatory proteins and their induced complement activation was reduced, suggesting a method to overcome hyperacute rejection and prolong the duration of graft survival [130]. This method improves the success rate of the pig-to-non-human primate xenotransplantation model. Additionally, due to the incompatibility of the coagulation system between pigs and primates, organ transplantation can lead to fibrin deposition and platelet aggregation between the tissues of the graft, resulting in intravascular thrombosis and ischemic injuries within the graft [115,130,131]. Transgenic pigs that express primate genes, including thrombomodulin, endothelial protein C receptor, complement-regulatory protein, CD46 (a membrane cofactor protein), CD55 (a decay-accelerating factor), and CD59 (a membrane attack complex inhibitor protein), have been developed to overcome these difficulties and enable the pig-to-non-human primate heart xenotransplantation model and future human heart xenotransplantation [128,132,133]. In addition to gene modification of the donor porcine, identifying effective immunosuppressive therapies and treating recipients will increase the success rate of xenotransplantation models. Antithymocyte globulin antibodies and anti-CD20 monoclonal antibodies are often used to reduce the function of T cells and B cells, respectively, reducing the occurrence of acute rejection [115,134,135,136]. Anti-CD40 monoclonal antibody blocks the CD40/CD154 pathway and prevents the T cell response, prolonging graft survival [128,134,135,136]. Conventional drugs, including mycophenolate mofetil, steroids, cyclosporine, and tacrolimus, are administered as maintenance therapy and to improve graft survival after xenotransplantation [115,137].

The α1,3-galactosyltransferase gene knockout/CD46 and thrombomodulin transgenic pig-to-non-human primate non-working heterotopic xenotransplantation model has been stable for over 2 years [115,126]. It has become a stable experimental animal platform for studying heart transplantation and xenograft development. The working heterotopic/orthotopic pig-to-non-human primate heart transplantation model is less commonly used in research than the non-working heterotopic xenotransplantation model due to the frequent occurrence of early graft failure and the insufficient duration of graft survival for long-term observation [115,128]. As the porcine donor heart is highly sensitive to I/R injury and frequent arrhythmias, graft failure due to humoral rejection may lead to early model failure [138]. An encouraging report of an orthotopic pig-to-primate xenotransplantation model indicates the potential for the clinical use of a pig heart. The model extends the maximum graft survival from 57 to 195 days. This model uses α-1,3-galactosyltransferase gene knockout/CD46 and thrombomodulin transgenic pig-to-non-human primate orthotopic xenotransplantation supplemented with antithymoglobulin antibodies, anti-CD20 monoclonal antibodies, mycophenolate mofetil, steroids, cyclosporine, and tacrolimus to ensure that the duration of the survival of the heart graft is extended [139].

In 2021 and 2022, researchers at the Langone Transplant Institute in New York University, USA and University of Maryland Medical Center, USA, transplanted hearts and kidneys from genetically modified pigs into humans. In 2021, Dr. Rober A. Montgomery et al. transplanted α-1,3 galactosyl-transferase-knockout pig kidney (provided by Revivicor Inc., Blacksburg, VA, USA) into patients determined to be brain-dead but were still on artificial ventilators to maintain physiological function and conducted two experiments [140]. Within 54 h of transplantation, the patient had normal renal function parameters and no hyperacute or acute rejection, demonstrating the feasibility of using genetically modified pigs for organ transplantation. Furthermore, with the support of this research, Dr. Nader Moazami of the Langone Transplant Institute at New York University successfully transplanted the hearts of 10 genetically modified pigs into two brain-dead patients. In January 2022, Dr. Bartley Griffith of the University of Maryland Medical Center in the United States transplanted a genetically modified pig heart into a patient with end-stage heart disease and severe arrhythmia, who was ineligible for human organ transplantation. After surgery, the patient was successfully disconnected from the ventilator and extracorporeal membrane oxygenation, but diastolic thickening developed 49 days after transplantation, and the patient died 60 days after transplantation [141]. The pathological analysis of the graft showed scattered myocyte necrosis, interstitial edema, and infiltration of red blood cells. However, there was no evidence of microvascular thrombosis, which is a typical sign of rejection. The research team led by Dr. Bartley Griffith will continue to analyze the real causes of graft failure. The application of CRISPR/Cas9 gene-editing technology to overcome rejection reactions and strict monitoring of cross-species infectious diseases make xenotransplantation a potential solution to address end-stage organ failure in the future [142].

## 4. Conclusions

Heart transplantation research in various animal models has advanced tremendously over the years. Animal models have been crucial in developing and refining heart transplantation techniques, studying the immune response to transplanted organs, and testing new immunosuppressive therapies. Establishing stable and controllable animal models is essential for studying the pathological mechanisms of organ transplantation and the development of xenografts. However, different animal models have provided significant contributions at different stages of organ transplantation and graft development studies. The earliest heart transplantation studies were performed in dogs in the 1950s and 1960s. These studies provided the first evidence that heart transplant from one animal to another was possible and that immunosuppressive therapy could reduce the risk of rejection. The dog model was instrumental in developing the surgical techniques and postoperative management protocols required for successful heart transplantation. In the 1970s, the pig model was introduced as a more practical alternative to the dog model. Pigs have human-like anatomy and physiology, and their larger size makes surgical procedures and monitoring easier. Pig models have been used to test new immunosuppressive therapies, improve surgical techniques, and develop new techniques for preserving donor hearts. Non-human primates have also been used extensively in heart transplantation research. Non-human primates have a more human-like immune system, and their usage has led to significant advancement in understanding the immune response to transplanted organs and the development of new immunosuppressive therapies. However, because of ethical considerations and the high cost of research, the use of non-human primates is controversial. Small-animal models, including rodents, rabbits, and guinea pigs, have also been used in heart transplantation research. These models have advantages, including high metabolic rates, high reproductive rates, small size for easy handling, and low cost. However, these models do not fully recapitulate the human immune system, and their use is limited to specific aspects of heart transplantation research, such as studying the mechanisms of graft rejection and testing new immunosuppressive therapies.

Overall, heart transplantation research in various animal models has advanced significantly over the years, providing valuable insights into the immunological and surgical aspects of the procedure. Continued breakthroughs in animal models will likely lead to further advancements in heart transplantation techniques and improved outcomes for patients in need of a heart transplant.

## Figures and Tables

**Table 1:** Small-animal/rodent models.

Specific Research Objectives	Methods	Materials	Advantages	Limitation	References
Antibody mediated rejection	Donor antigen-reactive monoclonal Abs injection	IgG2 and IgG1 Abs (anti-B10 Ab) to C57BL/6 mouse (with B10. A mouse heart)	Specific pathway of Ab mediated rejection	1. Do not induce enough rejection response 2. Research of abs class II MHC are limited	[4]
		IgG2 and IgG1 Abs (anti-Gal Ab) to C57BL/6 mouse (with Lewis rat heart)			[5]
	Donor antigen-reactive polyclonal Abs injection	polyclonal Abs injection	Adequate response of rejection	Multiple pathways for Ab-mediated rejection	[6]
	Sensitized serum-induced rejection	C57BL/6 *RAG1*^−/−^ with A/J heart injected with A/J sensitized mice serum	Adequate response of rejection		[7]
Hyperacute rejection	Rat-to-mouse abdominal xenotransplanted model	Lewis baby rat’s heart (αGal expressing) transplanted to C57BL/6 (αGal as the target Ag for rejection)			[5,8,9]
	Allograft transplantation across donor and recipient with or without expressing αGal	Transplant 129/C57BL/6 (αGal expressing) heart to C57BL/6			[10]
		Transplant BALB/c *αGal*^+/+^ heart to BALB/c *αGal*^−/−^ (pre-sensitized, expressing anti-αGal antibody)	Provide clear mechanism of interaction between αGal epitope and anti-αGal Abs		[11,12]
Graft accommodation	Rat-to mouse abdominal xenotransplanted model	Transplant baby Lewis rat heart to Rag/GT-deficient mice then inject anti-α-Gal IgG			[8]
Allograft tolerance	Induce immune regulatory response by immune pathway inhibitor and immune regulatory cells	Graft recipient injected with donor splenic leukocytes and B7/CD154 pathway inhibitor	1. Extended use of the model in immune regulatory studies 2. Accessible for large animal model		[13,14,15]
	Induce immune compromise by lymphocyte depletion	Injected alemtuzumab (anti-CD52 Abs) to human CD52 transgenic mouse			[16,17]
Regulating allograft rejection using skin transplantation	Heart and skin allograft transplantation across minor mismatched histocompatibility Ags	Female C57BL/6 first transplant with male C57BL/6 heart then male C57BL/6 skin			[18]
		Transplant BALB.B heart and skin to C57BL/6			[19]
		Non-vascularized ear–pinna cardiac allograft transplantation model			
	Heart and skin allograft transplantation across MHC disparities	Class II MHC mismatched donor skin transplanted to recipient with long-term-surviving heart allograft (class II MHC mismatched)			[20,21]
		Injected C57BL/6 immune cells (sensitized by A/J skin) to C57BL/6 (recipient of A/J heart)			[20]
Cardiac allograft vasculopathy	Combined abdominal heterotopic heart and aorta/carotid artery transplantation	Replace recipient’s left internal carotid artery with donor carotid artery and perform heterotopic abdominal heart transplantation in the same time	1. Better way to simulate the environment-generating allograft vasculopathy		[22]
		Heterotopic abdominal heart and aorta transplantation			[23]
	Heterotopic or orthotopic aortic transplantation	Brown-Norway (BN, RT1n) rat to Lewis (RT11) rat		Lack of immune response induced by parenchyma of transplanted graft that will not translate results well into the allograft vasculopathy involving a whole harvested heart.	[24]
		Lewis (RT11) rat to Fischer (RT11) rat			
		DA (RT1a) rat to Wistar–Firth (WF, RT1a) rat			
		PVG/Seac (RT1c) rat to AC I/NKyo (RT1a) rat.	1. Weak anti-donor rejection that is a better model for researching allograft vasculopathy 2. model which can be easily established and manipulated		[25,26,27]

**Table 2:** Models of innate immune response in heart transplantation for the roles of toll-like receptors and damage-associated molecular patterns.

Specific Research Objectives	Donor	Recipient	Target	References
Roles of toll-like receptors and damage-associated molecular patterns	coronary vessel ligation	C57BL/10 ScCr mouse C3H/HeJ mouse	TLR4	[37]
	coronary vessel ligation	C57Bl6 *TLR2*^−/−^ mouse	TLR2	[37]
	C57BL/6 *TLR4*^−/−^ mouse	C57BL/6 mouse	Hmgb1-TLR4-IL-23-IL-17A Axis	[38]
	C57BL/6 mouse	C57BL/6 mouse		
	BALB/c mouse	C57BL/6 mouse	Hmgb1	[39]
	BALB/c *MyD88*^+/+^ mouse BALB/c *MyD88*^−/−^ mouse	C57BL/6 *MyD88*^+/+^ mouse C57BL/6 *MyD88*^−/−^ mouse	myD88	[40]

**Table 3:** Models of innate immune in heart transplantation for the roles of antigen-presenting cell cells.

Specific Research Objectives	Donor	Recipient	Target	References
Roles of monocytes-macrophages, neutrophils, and NK cells	C57BL/6 mouse	(C57BL/6 mouse x BALB/c mouse) F1	Macrophages	[51]
	BALB/c mouse	C57BL/6 *RAG1*^−/−^ mouse	Monocytes	[52]
	BALB/c mouse	LysM^Cre^Traf6^fl/fl^ C57BL/6 mouse LysM^Cre^Mtor^fl/fl^ C57BL/6 mouse	TRAF6 or mTOR	[53]
	BALB/c mouse	LysM^Cre^Mtor^fl/fl^ C57BL/6 mouse	PD-1/PD-L1	
	BALB/c mouse	non-CSF1 expression C57BL/6 mouse	Macrophage	[54]
	BALB/c mouse	Fucci transgenic mice	Ly6C^lo^ macrophages	
	BALB/c mouse	C57BL/6 CD169 diphtheria toxin (DT) receptor (DTR) recipient mice	mTOR	[55]
	C3H and C57BL/6 mouse	BALB/c mouse	Mregs	[56]
	B6.129P2-*Nos2^tm/Lau^*/J mouse	BALB/c mouse	Mregs	
	A/J mouse	CXCR2-antisera–treated C57BL/6 mouse C57BL/6 *CXCR2*^−/−^ recipient	PMNs	[57]
	A/J mouse	C57BL/6 treated with anti-PMN Abs, KC/CXCL1 and MIP-2/CXCL2 Abs	PMNs	
	C57BL/6 mouse	(C57BL/6 mouse x BALB/c mouse) F1, (C57BL/6 mouse x B10.D2 mouse) F1, (C57BL/6 mouse x C3H/HeJ mouse) F1	NK cells	[58]
	B10.BR mouse	B57BL/6 *RAG1*^−/−^ mouse	NK cells	[59]

**Table 4:** Models of alloimmunity determined by major histocompatibility complex.

Specific Research Objectives	Donor	Recipient	Mismatched MHC Antigen	References
Complete MHC disparate	A/J mouse	129 mouse and C57BL/10 mouse	H-2K, H-2D, and multiple non-H-2	[28]
	A/J mouse	C57BL/6 mouse		[20,71]
	C3H mouse	(C57BL/10 mouse × DBA/1 mouse) F1		[71]
Single MHC disparate with class I MHC mismatch	B10.D2 mouse	(C57BL/6 mouse × A/J mouse) F1	H-2K	[28]
	B10.BR mouse	(C57BL/6 mouse × A/J mouse) F1	H-2D	
	BALB/c mouse (Ld-expressing)	C57BL/6 (Rag1 gene knockout) with Ld-reactive CD8 T cells	H-2Ld	[71]
	BALB/c (H-2dm2) mouse	C57BL/6 mouse	MHC Ⅰ + MHC II antigen without H-2Ld	[71]
	BALB/c mouse	C57BL/6 mouse	MHC Ⅰ + MHC Ⅱ antigen	
	C57BL/6 mouse (Kd-expressing)	C57BL/6 mouse	H-2Kd	[72]
Single MHC disparate with class II MHC mismatch	B6C.H-*2^bm12^* mouse	C57BL/6 mouse	1-A	[73,74]
Minor mH-Ags disparate	129/J mouse	C57BL/10 mouse	multiple non-H-2	[28]
	BALB.B mouse	C57BL/6 mouse	mH-Ags	[19]
	B10.D2 mouse	BALB/c mouse	mH-Ags	[75]
	male C57BL/6 mouse	female C57BL/6 mouse	mH-Ags	[9]

**Table 5:** Large-animal models.

Specific Research Objectives	Methods	Materials	Advantages	Limitation	References
Orthotopic heart transplantation	Bi-atrial anastomotic technique	Male Yorkshire pig donors and recipients	A more reliable pre-clinical model for researching rejection mechanisms and possible treatment		[111,112,113]
Non-working heterotopic heart transplantation	Recipient neck as the transplant site	Dog-to-dog heart transplantation	1. Easy handling 2. No drainage tube is needed 3. No post-surgical bowel obstruction 4. Graft’s condition easily evaluated by biopsy 5. Mild inflammatory response	1. The donor graft should be limited to under a certain volume due to the restricted space of the transplant site 2. Graft compresses recipient’s pharynx	[114,115]
		Cynomolgus monkey-to-baboon heart transplantation			[116]
	Recipient abdomen as the transplant site	Dog-to-dog heart transplantation	1. Larger space in transplant size 2. No limitation in graft size	1. High mortality rate in biopsy of the graft 2. Higher occurrence rate of ileus, intestinal obstruction, and intussusception	[117]
		Taiwanese Lanyu miniature pig heart transplantation			[118]
	Recipient retroperitoneum as the transplant site	Yorkshire piglet to baby baboon heart transplantation	1. Bigger space in transplant size 2. No limitation in graft size	Higher occurrence rate of postoperative ileus, intestinal obstruction, and intussusception	[119]
	Recipient thorax as the transplant site	Outbred mongrel dog heart transplantation		Chest tube drainage after surgery	[120]
Working heterotopic heart transplantation	Canine intrathoracic heterotopic heart transplant model	Dog-to-dog	Persistent systoling donor left ventricle with working load		[121]
	Biventricular working model	Heart transplant model using chacma baboon paired with ABO blood group	Working left and right ventricles		[122]
		Yorkshire pig heart transplantation			[123]

## Data Availability

Data sharing not applicable.
